# Differentiation of quantitative CT imaging phenotypes in asthma versus COPD

**DOI:** 10.1136/bmjresp-2017-000252

**Published:** 2017-11-09

**Authors:** Sanghun Choi, Babak Haghighi, Jiwoong Choi, Eric A Hoffman, Alejandro P Comellas, John D Newell, Sally E Wenzel, Mario Castro, Sean B Fain, Nizar N Jarjour, Mark L Schiebler, R Graham Barr, MeiLan K Han, Eugene R Bleecker, Christopher B Cooper, David Couper, Nadia Hansel, Richard E Kanner, Ella A Kazeroni, Eric A C Kleerup, Fernando J Martinez, Wanda K O’Neal, Prescott G Woodruff, Ching-Long Lin

**Affiliations:** 1 Department of Mechanical Engineering, Kyungpook National University, Daegu, South Korea; 2 Department of Mechanical and Industrial Engineering, University of Iowa, Iowa City, Iowa, USA; 3 IIHR-Hydroscience and Engineering, University of Iowa, Iowa City, Iowa, USA; 4 Department of Radiology, University of Iowa, Iowa City, Iowa, USA; 5 Department of Internal Medicine, University of Iowa, Iowa City, Iowa, USA; 6 Division of Pulmonary, Allergy and Critical Care Medicine, University of Pittsburgh, Pittsburgh, Pennsylvania, USA; 7 Departments of Internal Medicine and Pediatrics, Washington University School of Medicine, St. Louis, Missouri, USA; 8 Departments of Radiology and Medicine, School of Medicine and Public Health, University of Wisconsin, Madison, Wisconsin, USA; 9 Department of Medical Physics and Biomedical Engineering, University of Wisconsin, Madison, Wisconsin, USA; 10 Mailman School of Public Health, Columbia University, New York, USA; 11 Department of Internal Medicine, University of Michigan, Ann Arbor, Michigan, USA; 12 Center for Genomics and Personalized Medicine, Wake Forest University, Winston-Salem, North Carolina, USA; 13 Department of Physiology, University of California, Los Angeles, California, USA; 14 Department of Global Public Health, University of North Carolina, Chapel Hill, North Carolina, USA; 15 School of Medicine, Johns Hopkins University, Baltimore, Maryland, USA; 16 School of Medicine, University of Utah, Salt Lake City, Utah, USA; 17 Department of Radiology, University of Michigan, Ann Arbor, Michigan, USA; 18 Department of Medicine, University of California, Los Angeles, Los Angeles, California, USA; 19 Department of Medicine, Weill Cornell School of Medicine, Cornell University, New York, USA; 20 Marsico Lung Institute, University of North Carolina, Chapel Hill, North Carolina, USA; 21 School of Medicine, University of California at San Francisco, San Francisco, California, USA

**Keywords:** emphysema, functional small airway disease, image registration, quantitative computed tomography, airway luminal narrowing

## Abstract

**Introduction:**

Quantitative CT (QCT) imaging-based metrics have quantified disease alterations in asthma and chronic obstructive pulmonary disease (COPD), respectively. We seek to characterise the similarity and disparity between these groups using QCT-derived airway and parenchymal metrics.

**Methods:**

Asthma and COPD subjects (former-smoker status) were selected with a criterion of post-bronchodilator FEV_1_ <80%. Healthy non-smokers were included as a control group. Inspiratory and expiratory QCT images of 75 asthmatic, 215 COPD and 94 healthy subjects were evaluated. We compared three segmental variables: airway circularity, normalised wall thickness and normalised hydraulic diameter, indicating heterogeneous airway shape, wall thickening and luminal narrowing, respectively. Using an image registration, we also computed six lobar variables including per cent functional small-airway disease, per cent emphysema, tissue fraction at inspiration, fractional-air-volume change, Jacobian and functional metric characterising anisotropic deformation.

**Results:**

Compared with healthy subjects, both asthma and COPD subjects demonstrated a decreased airway circularity especially in large and upper lobar airways, and a decreased normalised hydraulic diameter in segmental airways. Besides, COPD subjects had more severe emphysema and small-airway disease, as well as smaller regional tissue fraction and lung deformation, compared with asthmatic subjects. The difference of emphysema, small-airway disease and tissue fraction between asthma and COPD was more prominent in upper and middle lobes.

**Conclusions:**

Patients with asthma and COPD, with a persistent FEV_1_ <80%, demonstrated similar alterations in airway geometry compared with controls, but different degrees of alterations in parenchymal regions. Density-based metrics measured at upper and middle lobes were found to be discriminant variables between patients with asthma and COPD.

**Key messages**

- We demonstrate similarity and disparity of structure and function between patients with asthma and chronic obstructive pulmonary disease (COPD) with chronic functional alteration using sensitively measured imaging metrics.
- Image-derived functional alterations of asthmatics were regionally prominent in lower lobes, while those of patients with COPD were found in whole lungs.

## Introduction

Asthma is a disease with functional airway reversibility through the aid of inhaled corticosteroids and bronchodilator, whereas chronic obstructive pulmonary disease (COPD) is a disease with persistent airflow limitation.^1–3^ According to recent reports,^4 5^ around 15%–45% of patients with COPD may have asthma–COPD overlap, so-called ACO. In previous studies,^6 7^ some asthmatic patients were of neutrophilic dominance with chronic airway functional alteration, while some patients with COPD were of eosinophilic dominance with airway reversibility.^8 9^ Thus, objective differentiation of the two populations is essential for proper treatments. Meanwhile, two National Institutes of Health (NIH)-supported multicentre studies, SubPopulations and InteRmediate Outcome Measures In COPD Study (SPIROMICS)^10^ and Severe Asthma Research Program (SARP),^11^ have been established to identify genetic, environmental and clinical phenotypes for COPD and asthma, respectively. SARP excluded patients with a smoking history, while SPIROMICS, except healthy subjects, excluded never-smokers. By the study designs, the recruited subjects are less likely to be ACO. With these populations, we pursue to investigate the imaging-based similarity and disparity between two respiratory diseases.

Quantitative CT (QCT) imaging has successfully identified unique structural and functional phenotypes for asthma and COPD, respectively. For instance, Aysola *et al* 12 found that asthmatics had an increase of wall area percentage depending on severity, and Busacker *et al* 13 found an increase of air trapping in severe asthmatics. Using the same imaging datasets from SARP, Choi *et al* 14 15 demonstrated that existing imaging-based metrics were problematic due to intersubject and intersite variability, resulting in inaccurate estimation. To address these issues, they developed a new air-trapping measure and new normalisation schemes for luminal hydraulic diameter and wall thickness.^14 15^ These studies demonstrated that on average severe asthmatics were characterised by airway narrowing, wall thickening and air trapping. In addition, with an image registration technique, they demonstrated regional alterations of deformational metrics in severe asthmatics.^16^ The sensitive imaging-based metrics derived from multiple studies were then integrated to identify clinically meaningful subgroups of asthma.^17^

In patients with COPD, structural and functional alterations have been assessed by QCT imaging-based variables including luminal diameter,^18^ wall thickness,^19^ air trapping (or functional small-airway disease) and emphysema. Existing assessment of air trapping in COPD was also problematic because air trapping at expiration contains some portion of emphysema at inspiration. Therefore, Galban *et al* 20 employed an image registration technique to dissociate the portion of emphysema from air trapping, allowing for characterisation of three subgroups, that is, emphysema-dominant, functional small-airway disease-dominant and normal groups. In addition to the CT density mapping, image registration provided local deformational metrics including air-volume change, the determinant of Jacobian (Jacobian; a measure of volume change), anisotropic deformation index (ADI; a measure of the magnitude of directional preference in volume change), slab rod index (a measure of the nature of directional preference in volume change) and more.^21^ Bodduluri *et al* 22 used the image registration metrics to perform a supervised learning for the purpose of distinguishing patients with COPD from non-COPD subjects. Further, Smith *et al* 19 compared wall thicknesses of patients with COPD and non-COPD subjects, and found thinner airway walls in patients with COPD than non-COPD subjects when wall thickness is compared at the same location.

QCT imaging-based variables have been employed in a variety of studies to identify local or global alterations in airway dimension and lobar function. Although there are several studies to compare airway structure or lung function between asthma and COPD,^23–25^ the numbers of subjects under investigation were limited. Therefore, this study aims to investigate the similarity and disparity between large cohorts of asthma and COPD subjects acquired from SARP and SPIROMICS, especially those with overlapping clinical symptoms of chronic functional alterations measured by post-bronchodilator FEV_1_ <80%. In order to assess structure and function at global and lobar levels, we employed multiscale imaging-based variables,^14–17^ including local airway structural variables at inspiration scan, and lobar/global functional variables at expiration scan. We further employed image registration metrics including Jacobian, ADI and functional small-airway disease percentage (fSAD%) and emphysematous lung percentage (Emph%).

## Methods

### Inclusion criteria and QCT imaging data

We employed QCT imaging data from SARP and SPIROMICS. Both populations used a similar imaging protocol which has been described by Sieren *et al* 26 and were approved by the respective institutional review boards. We selected subjects who demonstrated post-bronchodilator FEV_1_ %predicted values <80% to focus on subjects with airflow obstruction. In SARP data, none of the asthmatics were current smokers as one of the inclusion criteria was ‘no smoking within the past five years and <5 pack-years of smoking’.^27^ SPIROMICS, except for healthy controls, excluded non-smokers, and in this study only former smokers were included using the criterion of ‘not currently smoking as of 1 month ago’.^10^ The numbers of asthmatic and COPD subjects meeting these criteria were 75 and 215. In addition, 94 healthy subjects without smoking history including 51 SARP and 43 SPIROMICS subjects were used as a control group (table 1). There are more imaging data in SARP and SPIROMICS, but the imaging data from eight SPIROMICS and three SARP centres were only used in this study (table 2). The imaging data from SARP were previously employed to identify imaging-based asthma clusters.^17^ All of the imaging centres acquired inspiratory and expiratory QCT scans for each subject. While both SPIROMICS and SARP obtained inspiration scans at TLC, expiration scans were obtained at functional residual capacity (FRC) for SARP and residual volume (RV) for SPIROMICS. Thus, caution must be taken in interpreting results associated with expiration scan and volumetric difference. For instance, fSAD% and Jacobian may increase and decrease respectively by using FRC rather than RV scans.

**Table 1:** Demographic, pre-bronchodilator and post-bronchodilator pulmonary function tests, blood inflammatory information among healthy subjects, patients with asthma and patients with chronic obstructive pulmonary disease (COPD)

	Healthy subjects (n=94§)	Patients with asthma (n=75)	Patients with COPD (n=215)
Demography			
Age (years)	50.7 (17.2)	48.7 (11.2)	67.1 (7.2)†‡
Female sex (%)	59	47	41
Body mass index (kg/m^2^)	27.7 (5.5)	33.5 (7.5)*	27.9 (4.9)†
Race (white/African-American/other) (%)	65/20/15	76/17/7	87/7/6‡
Pre-bronchodilator			
FEV_1_, %predicted	98 (12)	53 (14)*	44 (18)†‡
FVC, %predicted	98 (10)	69 (13)*	76 (17) †‡
FEV_1_/FVC	79 (6)	61 (11)*	43 (13)†‡
Post-bronchodilator			
FEV_1_, %predicted	103 (11)	65 (11)*	51 (19)†‡
FVC, %predicted	99 (10)	80 (14)*	85 (18)‡
FEV_1_/FVC	82 (6)	65 (11)*	45 (13)†‡
Blood tests			
Samples (n)	93	73	213
Total white blood cells (N/μL)	5894 (1717)	7902 (2962)*	7247 (1999)‡
Neutrophils (%)	58.1 (7.8)	59.2 (12.4)	63.3 (9.7)‡
Lymphocytes (%)	31.7 (7.4)	28.2 (10.5)	24.6 (8.3)‡
Eosinophils (%)	2.3 (1.6)	3.9 (3.7)*	2.9 (1.8)‡

*P<0.01 for healthy versus asthmatic subjects.

†P*<*0.01 for asthmatic versus COPD subjects.

‡P*<*0.01 for COPD subjects versus healthy subjects.

§Healthy datasets contain 51 Severe Asthma Research Program and 43 SubPopulations and InteRmediate Outcome Measures In COPD Study subjects. Benjamini–Hochberg post-hoc tests were performed.

FEV1, Forced expiratory volume in one second; FVC, Forced vital capacity.

**Table 2:** The number of quantitative CT imaging datasets according to imaging centres: each dataset contains one inspiration and one expiration scan

Project	Imaging centre (abbreviation)	Number of datasets
SubPopulations and InteRmediate Outcome Measures In COPD Study (eight sites)		284
	Columbia University (CU)	61
	Johns Hopkins University (JH)	24
	University of California at Los Angeles (LA, LV)	36, 15 (two sites)
	University of Michigan (MU)	39
	University of California at San Francisco (SF)	31
	University of Utah (UT)	59
	Wake Forest University (WF)	19
Severe Asthma Research Program (three sites)		100
	University of Pittsburgh (PIT)	44
	University of Wisconsin (WIS)	38
	Washington University in Saint Louis (WSL)	18

### An expanded set of multiscale imaging-based variables

We employed a total of 67 imaging-based metrics, where 55 of them were multiscale imaging-based metrics developed for the asthma cluster analysis.^17^ Using Apollo (VIDA Diagnostics),^28^ we derived three local structural variables: airway circularity (*Cr*), wall thickness (WT) and hydraulic diameter (*D*_h_) extracted at total lung capacity (TLC) scans. WT and *D*_h_ were normalised with the tracheal WT and *D*_h_ predicted from healthy subjects, denoted as WT* and *D*_h_*.^14^ *Cr*, WT* and *D*_h_* indicate heterogeneous airway shape, airway wall thickening and airway luminal narrowing, respectively. A detailed description of airway-structural imaging metrics can be found in ref.^14^ Furthermore, for the comparison with patients with COPD, we used the parametric response map^20^ to subtract the emphysematous lung regions from regions labelled as air trapped on expiratory scans using an image registration technique,^29^ being denoted as fSAD%. We also added two more variables: Emph% and tissue fraction at TLC (β_tissue_). Rather than the CT density-threshold method, we used a fraction-threshold method to determine fSAD% (air fraction, β_air_ >90%) and Emph% (β_air_ >98.5%), eliminating intersite variability.^15^ Next, β_tissue_ was computed by the portion of tissue volume in a voxel to evaluate an alteration of local tissue. Furthermore, we computed fractional lobar air-volume change (ΔV_air_^f^), Jacobian and ADI via image registration.^16 17^ ΔV_air_^f^, Jacobian and ADI indicated preferential lobar air-volume change (a measure of regional ventilation), local expansion (a measure of regional volume change) and non-uniform stretch (a measure of the magnitude of direction preference in volume change), respectively, in parenchymal regions. A detailed description of functional imaging metrics is available in refs.^16 17^

### Statistical analysis

Kruskal-Wallis and χ^2^ test^30^ were performed to compare differences of continuous and categorical variables between healthy, asthmatic and COPD subjects. We performed a total of 87 comparison tests, and a P value of 0.01 was taken as the significant level in all tests. This controls the false discovery rate of multiple comparisons at 1%, as estimated by using the method of Benjamin and Hochberg.^31^

## Results

### Demography, PFTs and blood tests

Table 1 shows demography, pre-bronchodilator and post-bronchodilator pulmonary function test (PFT) and blood test results among three populations, that is, healthy subjects, patients with asthma and patients with COPD. Ages of patients with COPD were greater than those of healthy and asthmatic subjects, and the portion of female sex was similar between asthma (47%) and COPD (41%). Asthmatic patients were more obese than healthy subjects and patients with COPD, whereas there was a lower portion of African-Americans in COPD. Compared with healthy subjects, both asthmatic patients and patients with COPD had lower FEV_1_ and FVC %predicted values according to their inclusion criteria with FEV_1_ %predicted value <80%. Pre-bronchodilator FEV_1_ %predicted values of patients with COPD were lower than those of asthma, while FVC % predicted values of patients with COPD were greater than those of asthma, leading to a further reduction of FEV_1_/FVC in patients with COPD. Thus, at pre-bronchodilator, flow restriction and obstruction were dominant for asthma and COPD, respectively. At post-bronchodilator, the difference between FEV_1_ %predicted values for asthma and COPD were more significant, whereas FVC %predicted values were close for both populations due to the functional reversibility of asthma. We further compared blood inflammatory biomarkers. Both patients with asthma and COPD demonstrated greater numbers of white blood cell counts than those of healthy subjects. The percentages of neutrophils and lymphocytes increased and decreased in patients with COPD, respectively, compared with those of healthy and asthmatic subjects. The percentages of eosinophils in both asthma and COPD were elevated, relative to that of healthy subjects.

### Central airway-structural characteristics

**Figure 1:**
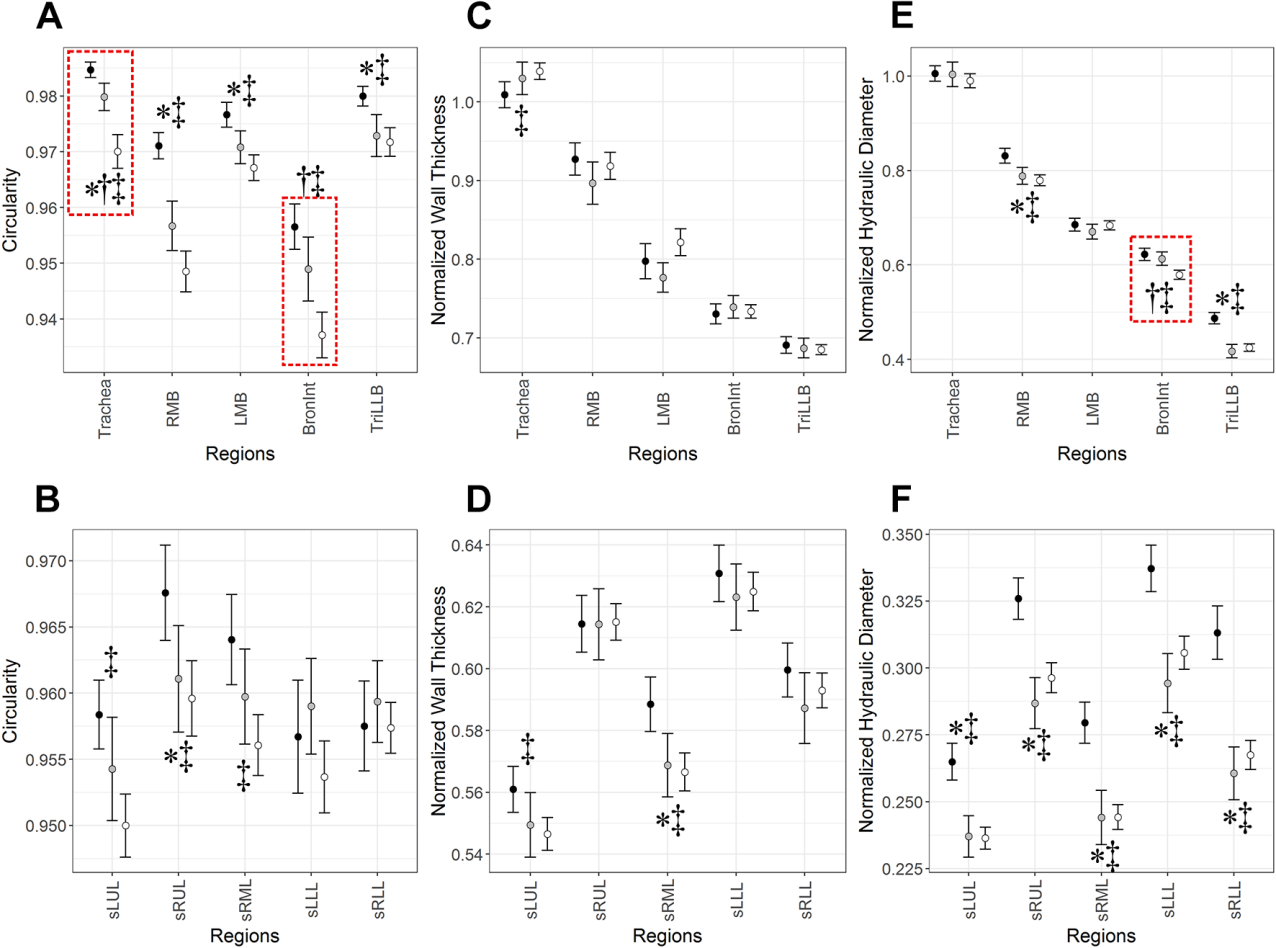
(A, B) Airway circularity (*Cr*), (C, D) normalised wall thickness (WT*) and (E, F) normalised hydraulic diameter (*D*_h_*) in 10 local regions among healthy, asthmatic and chronic obstructive pulmonary disease (COPD) subjects. Values are presented as mean (±CI). *P<0.01 for healthy versus asthmatic subjects. †P*<*0.01 with red-dashed box for asthmatic versus COPD subjects. ‡P*<*0.01 for COPD subjects versus healthy subjects. BronInt, right intermediate bronchus; LMB, left main bronchus; RMB, right main bronchus; sLLL, subgrouped left lower lobe; sLUL, subgrouped left upper lobe; sRLL, subgrouped right lower lobe; sRML, subgrouped right middle lobe; sRUL, subgrouped right upper lobe; TriLLB, trifurcation of left lower lobe.

Figure 1 shows regional difference of *Cr*, WT* and *D*_h_* among healthy, asthmatic and COPD subjects. For a better presentation, we divided 10 local branches into two regions (top vs bottom columns), that is, five central airways (trachea, right main bronchus (RMB), left main bronchus (LMB), right intermediate bronchus (BronInt) and trifurcation of left lower lobe (TriLLB)), and five segmental airways (subgrouped left upper lobe (sLUL), subgrouped right upper lobe (sRUL), subgrouped right middle lobe (sRML), subgrouped left lower lobe (sLLL) and subgrouped right lower lobe (sRLL)). *Cr* significantly decreased in both asthmatic patients and patients with COPD compared with healthy subjects especially in central airways, where COPD decreased more than asthma in trachea and BronInt (figure 1A). In segmental airways, *Cr* of healthy subjects versus patients with COPD was only different in upper and middle lobes (see sLUL, sRUL and sRML in figure 1B). WT* of COPD subjects increased only in trachea compared with healthy subjects (figure 1C), while it decreased in sLUL and sRML of patients with COPD (figure 1D). Other than these three regions, there was no noticeable difference of WT* among three populations. There was no difference of *D*_h_* in trachea and LMB, whereas *D*_h_* of patients with asthma and COPD significantly decreased in other regions except these two regions (figure 1E, F). The reduced quantities of *D*_h_* in segmental airways between asthma and COPD were similar.

### Parenchymal functional characteristics

**Figure 2:**
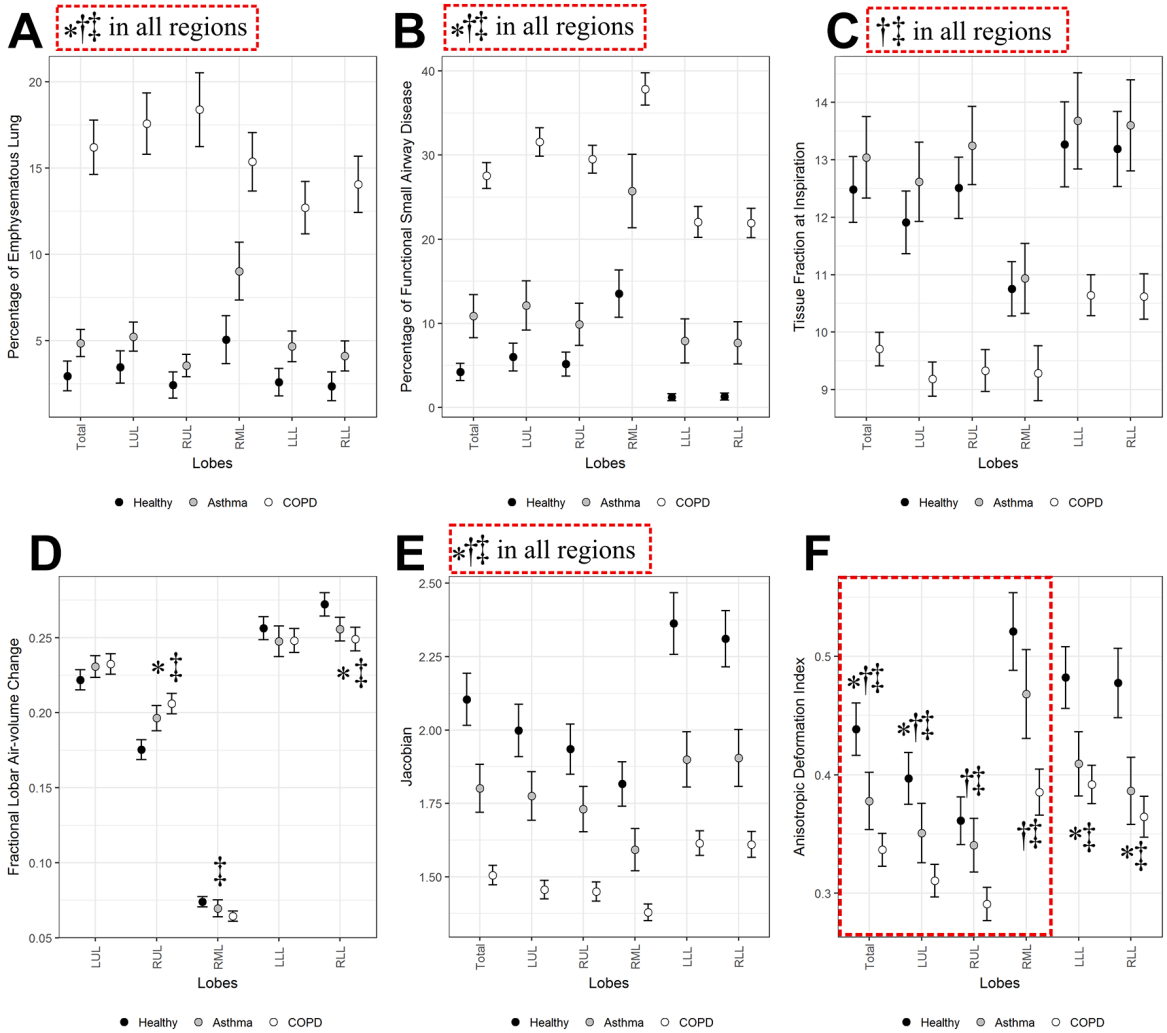
(A) Percentage of emphysematous lung (Emph%), (B) functional small airway disease (fSAD%), (C) tissue fraction at inspiration (β_tissue_), (D) fractional lobar air-volume change (Δ*V*_air_^f^), (E) the determinant of Jacobian (Jacobian) and (F) anisotropic deformation index (ADI) among healthy, asthmatic and chronic obstructive pulmonary disease (COPD) subjects. Values are presented as mean (±CI). *P<0.01 for healthy versus asthmatic subjects. †P*<*0.01 with red-dashed box for asthmatic versus COPD subjects. ‡P*<*0.01 for COPD subjects versus healthy subjects. LLL, left lower lobe; LUL, left upper lobe; RLL, right lower lobe; RML, right middle lobe; RUL, right upper lobe.

We also compared parenchymal/global functional metrics including Emph%, fSAD%, β_tissue_, Δ*V*_air_^f^, Jacobian and ADI (figure 2A–F). The former four variables were associated with CT density-based assessment, whereas the latter two variables were associated with mechanical strains estimated by image registration. As expected, Emph% of COPD subjects significantly increased compared with those of healthy and asthma subjects, and asthma subjects also showed a significant elevation of Emph% (figure 2A). fSAD% significantly increased in patients with COPD and asthma, especially in patients with COPD (figure 2B). Relative to healthy subjects, a new variable β_tissue_ at inspiration decreased in entire lungs in patients with COPD (figure 2C), possibly due to tissue destruction by emphysema. Unlike Emph%, β_tissue_ was similar between healthy and asthmatic subjects, whereas it decreased only in patients with COPD. Δ*V*_air_^f^ behaved similarly in both patients with asthma and COPD, such that Δ*V*_air_^f^ increased in RUL and it decreased in right lower lobe (RLL) (figure 2D). Thus the relative contribution of air-volume change was shifted from lower and middle lobes to upper lobes. Jacobian, indicating local volume change, decreased in both disease groups but more in patients with COPD (figure 2E). ADI, indicating preferential deformation, decreased more in upper lobes of COPD subjects, whereas it decreased similarly in lower lobes of patients with asthma and COPD (figure 2F). This may indicate difference of upper lobar characteristics in deforming lungs between patients with asthma and COPD.

## Discussion

Using QCT imaging-based variables, we have investigated the structural and functional characteristics at segmental and lobar/global scales between two airway obstructive diseases, for example, asthma and COPD. These subjects were clinically diagnosed with either asthma or COPD by NIH-sponsored multicentre studies of SARP and SPIROMICS. In this study, we compared currently non-smoking patients with asthma and former smoker patients with COPD with similar clinically overlapping characteristics of reduced lung function measured by post-bronchodilator FEV_1_ <80%. Asthma is characterised by airway narrowing, wall thickening and air trapping (or functional small-airway disease) and reduced lung deformation.^12–16^ COPD is characterised by airway narrowing, air trapping, increased emphysematous lung percentage and reduced lung deformation.^18–20 22^ As multicentre studies, SARP and SPIROMICS contributed to identify disease phenotypes by collecting subject demographics, biospecimens, QCTs and other data for patients with asthma and COPD. The present study has been able to take unique advantage of these multicentre studies with similar imaging protocols and similar image analysis, allowing for a comparison of the QCT data serving to define asthma and COPD imaging phenotypes. A better understanding of distinct structural and functional features between the two populations may help to develop personalised treatment intervention and improve the classification of asthma and COPD.

Regarding airway structure, *Cr*, WT* and *D*_h_* demonstrated different characteristics from observed features in healthy subjects. *Cr,* indicating the heterogeneous distribution of airway shape, was mostly altered in large airways and segmental bronchi in upper and middle lobes. COPD demonstrated the greater degree of these alterations relative to asthma (figure 2A, B). WT* seems to be clearly increased only in the trachea of patients with COPD compared with healthy patients and patients with asthma. The alterations of *Cr* and WT* in larger airways (or lower generations) may indicate that airway remodelling due to chronic inflammation or chronic bronchitis was predominantly caused in large airways rather than small airways. Unlike *Cr* and WT*, *D*_h_*, indicating airway narrowing, was significantly decreased in both asthma and COPD to a similar degree compared with healthy subjects. We have previously reported that among airway structural variables *D*_h_* was the most significantly correlated with PFT-based FEV_1_ and FEV_1_/FVC.^14^ Since we studied patients with lower FEV_1_, a smaller *D*_h_* was expected. With regard to regional phenotype, as shown in figure 2, airway structural alterations estimated by *Cr*, WT* and *D*_h_* were located randomly, implying that each metric demonstrates different phenotype.

Compared with airway structural variables, parenchymal functional variables provided much clearer differences between asthma and COPD. Although asthma demonstrated an increase of Emph%, which might be the result of unmeasurable environmental exposures.^32^ As would be expected, we found a significantly greater elevation in Emph% in COPD. Although fSAD% is an independent variable from Emph%, it exhibited a similar pattern with Emph%, such that patients with COPD had a greater elevation of fSAD% than asthma. While *D*_h_* had similar values between asthma and COPD, fSAD%, indicating small-airway disease, had significantly different values between asthma and COPD. This may indicate that small airway disease in patients with COPD is more prominent than asthmatic subjects. In other words, asthma reversibility is an effect predominantly localised to the peripheral airways as demonstrated by Verbanck *et al*.^33 34^ In this study, we also introduced a new variable β_tissue_ to evaluate the state of tissue alteration.^35^ A decrease of β_tissue_ is possibly related to tissue destruction due to emphysema. Thus, β_tissue_ can serve as a metric used to differentiate between asthma, COPD and healthy subjects in addition to Emph% and fSAD%.

Using an image registration technique, we derived Δ*V*_air_^f^, Jacobian and ADI. Δ*V*_air_^f^, indicating regional distribution of air-volume change, seemed to increase in RUL and decrease in RLL consistently in asthma and COPD subjects. The similar finding has been reported in our previous study with 30 severe asthmatics.^16 17^ Jacobian, indicating the degree of local deformation, was significantly reduced in both asthma and COPD, with a more severe reduction in COPD. ADI seemed to have different characteristics from Jacobian in that it decreased in lower lobes for both asthma and COPD. On the other hand, it seemed to be relatively normal in RUL and RML in asthmatics, unlike patients with COPD. Based on Δ*V*_air_^f^ and ADI, functional alterations of asthmatics were observed more in lower lobes, whereas those of COPD were observed in entire lungs.

When comparing multicentre data, it is necessary to use standardised imaging protocols. Since this study is designed retrospectively, there were protocol differences. The most critical difference between SARP and SPIROMICS is about lung volume when obtaining expiratory scans. SARP took expiratory scans at FRC, whereas SPIROMICS did this at RV. Thus COPD subjects obtained from SPIROMICS should have larger Jacobian and smaller fSAD% because RV is smaller lung volume than FRC. Nevertheless, the results showed that COPD subjects had significantly smaller Jacobian and larger fSAD% than asthmatic subjects which likely go beyond differences that would be expected from imaging at RV versus FRC. Therefore, the actual difference between asthma and COPD should be greater than that reported here if the same imaging protocol were used. It also should be noted that obesity in asthmatics may reduce expiratory reserve volume, making FRC closer to RV.^36^ Furthermore, due to the disease features, asthma is likely to be early onset, whereas COPD is likely to be late onset. Asthma is also associated with obesity, whereas COPD is with loss of weight. These disease features prevent from matching their demographics. Another issue may be raised due to intersubject variability including sex, age and height. We previously found that sex is the most important metric to determine airway size including hydraulic diameter and wall thickness; therefore, we developed new normalisation schemes to control the intersubject variability. Furthermore, there was no bias of samples due to the female sex between asthma and COPD (table 1).

In conclusion, QCT imaging-based variables were found to be effective in differentiating asthmatics from healthy subjects as well as COPD subjects from asthma subjects. Both asthma and COPD had a decrease of airway luminal circularity particularly in larger airways and a decrease of hydraulic diameter in segmental airways to the same degree. Functional metrics, especially density-based metrics, obtained at lobar/global regions were found to be significantly different between the two disease populations. Compared with asthma, COPD has significantly more lung emphysema, more small-airway disease, with reduced tissue fraction and regional lung deformation. Regionally, functional alterations of asthmatics were prominent in lower lobes, while those of patients with COPD were found in whole lungs. Based on these functional and regional features, density-based metrics including tissue fraction, fSAD% and Emph% measured at parenchymal levels were found to be important in differentiating patients with asthma and COPD.
