# Correction: *Differentiation of quantitative CT imaging phenotypes in asthma versus COPD*

**DOI:** 10.1136/bmjresp-2017-000252corr1

**Published:** 2018-03-06

Choi S, Haghighi B, Choi J, *et al*. Differentiation of quantitative CT imaging phenotypes in asthma versus COPD. *BMJ Open Resp Res* 2017;**4**:e000252. doi:10.1136/bmjresp-2017-000252.

The author’s name, Kazeroni EA, has been corrected to Kazerooni EA.

